# Clonal and microclonal mutational heterogeneity in high hyperdiploid acute lymphoblastic leukemia

**DOI:** 10.18632/oncotarget.12238

**Published:** 2016-09-24

**Authors:** Adam J. de Smith, Juhi Ojha, Stephen S. Francis, Erica Sanders, Alyson A. Endicott, Helen M. Hansen, Ivan Smirnov, Amanda M. Termuhlen, Kyle M. Walsh, Catherine Metayer, Joseph L. Wiemels

**Affiliations:** ^1^ Department of Epidemiology and Biostatistics, University of California San Francisco, San Francisco, California, United States of America; ^2^ Division of Neuroepidemiology, Department of Neurological Surgery, University of California San Francisco, San Francisco, California, United States of America; ^3^ Children's Hospital Los Angeles, Department of Pediatrics, Keck School of Medicine, University of Southern California, Los Angeles, California, United States of America; ^4^ School of Public Health, University of California Berkeley, Berkeley, California, United States of America

**Keywords:** high hyperdiploid acute lymphoblastic leukemia, targeted deep-sequencing, microclonal mutations, tumor heterogeneity, DOT1L

## Abstract

High hyperdiploidy (HD), the most common cytogenetic subtype of B-cell acute lymphoblastic leukemia (B-ALL), is largely curable but significant treatment-related morbidity warrants investigating the biology and identifying novel drug targets. Targeted deep-sequencing of 538 cancer-relevant genes was performed in 57 HD-ALL patients lacking overt *KRAS* and *NRAS* hotspot mutations and lacking common B-ALL deletions to enrich for discovery of novel driver genes. One-third of patients harbored damaging mutations in epigenetic regulatory genes, including the putative novel driver *DOT1L* (*n*=4). Receptor tyrosine kinase (RTK)/Ras/MAPK signaling pathway mutations were found in two-thirds of patients, including novel mutations in *ROS1*, which mediates phosphorylation of the *PTPN11-*encoded protein SHP2. Mutations in *FLT3* significantly co-occurred with *DOT1L* (*p*=0.04), suggesting functional cooperation in leukemogenesis. We detected an extraordinary level of tumor heterogeneity, with microclonal (mutant allele fraction <0.10) *KRAS*, *NRAS*, *FLT3*, and/or *PTPN11* hotspot mutations evident in 31/57 (54.4%) patients. Multiple *KRAS* and *NRAS* codon 12 and 13 microclonal mutations significantly co-occurred within tumor samples (*p*=4.8×10^−4^), suggesting ongoing formation of and selection for *Ras*-activating mutations. Future work is required to investigate whether tumor microheterogeneity impacts clinical outcome and to elucidate the functional consequences of epigenetic dysregulation in HD-ALL, potentially leading to novel therapeutic approaches.

## INTRODUCTION

High hyperdiploidy (HD), characterized by a non-random pattern of chromosomal gains (51 to 67 chromosomes), comprises the largest cytogenetic subgroup of B-cell acute lymphoblastic leukemia (B-ALL) and is one of the most common malignancies in children [1]. Although HD usually confers a favorable prognosis, survivors of childhood ALL have long-term treatment-related morbidity and mortality, including secondary cancers, cardiovascular disease and pulmonary disease [2, 3]. A fuller understanding of the genomic landscape driving leukemogenesis in HD-ALL may inform the biology of this disease and potentially reveal novel therapeutic strategies.

Hyperdiploidy has been shown to arise prenatally [4, 5] and is likely initiated by a single abnormal mitotic event [6, 7], implicating it as the primary genetic lesion. This is followed by additional somatic alterations, arising pre- or postnatally, and these are believed to be required for overt leukemia. Distinct characteristics of HD-ALL have been highlighted both by studies of heritable and acquired genetic alterations. Genome-wide association studies (GWAS) have identified SNPs that distinguish HD-ALL from other subtypes, for example in *ARID5B*, *CEBPE*, and *PIP4K2A* [8-11]. Similarly, a distinct pattern of somatic mutations characterizes HD-ALL. For instance, frequency of common childhood ALL gene deletions, including *CDKN2A*, *IKZF1*, and *PAX5*, is relatively low [12], whereas activating mutations in receptor tyrosine kinase (RTK)/Ras/MAPK signaling genes *KRAS*, *NRAS*, *FLT3*, and *PTPN11*, are relatively common [13-15]. Indeed, recent next-generation sequencing analyses of HD-ALL confirmed that mutations in these genes, and in the epigenetic regulatory gene *CREBBP*, are the most frequent somatic alterations in this leukemia subtype [16, 17]. Here, we describe targeted sequencing of 538 cancer-relevant genes using a clinical sequencing platform in a set of 57 HD-ALL patients lacking the common *KRAS* and *NRAS* codon 12 and 13 hotspot mutations and common gene deletions, to enrich for discovery of novel driver genes and to assess recurrently-mutated pathways in HD-ALL tumorigenesis. This deep-sequencing enabled identification of putative novel drivers and permitted an in-depth assessment of intratumoral heterogeneity.

## RESULTS

**Figure 1:**
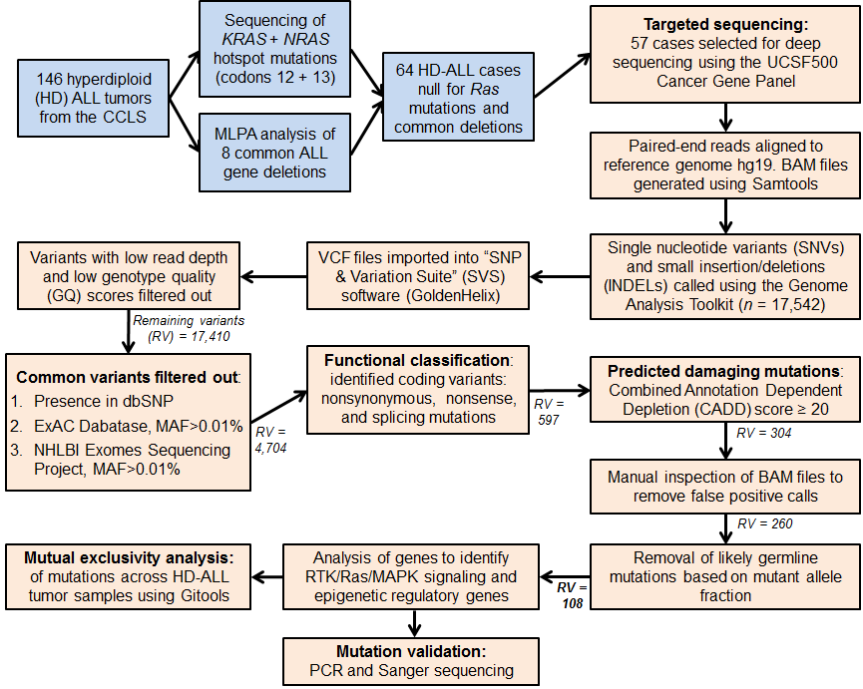
Flow diagram summarizing the analysis pipeline Blue colored boxes represent subject selection criteria. Peach colored boxes include sequencing data and variant filtering analyses. MAF refers to the SNP minor allele frequency. Remaining variants (RV) refers to the number of combined SNVs and INDELs remaining in the analysis pipeline following each filtering step. The final number of variants highlighted in bold (RV = 108) refers to the predicted damaging and likely somatic variants detected in the 57 HD-ALL patients.

**Figure 2:**
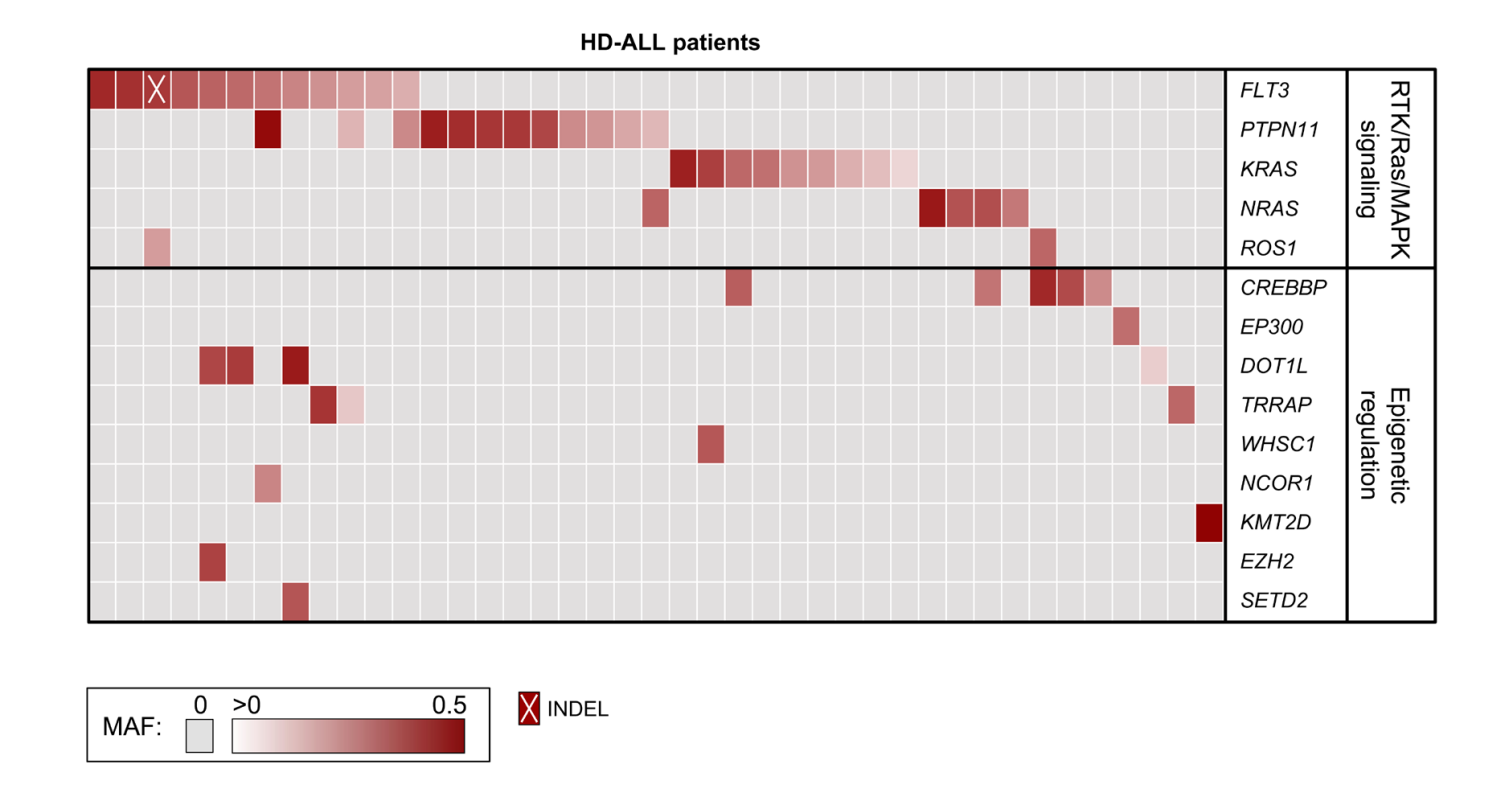
Tiling plot of mutations in high hyperdiploid ALL Predicted damaging mutations are included for recurrently affected genes and for genes previously identified as mutated in HD-ALL (rows) across 41 of 57 HD-ALL patients (columns) in the study. Patients without mutations in such genes were excluded from the figure. Mutations are color-coded according to their mutant allele fraction (MAF), and INDELs are highlighted as shown at the bottom of the figure. MAF was adjusted for chromosome copy number. Where > 1 mutation was present in the same gene in a patient, the color of the cell represents the most clonal mutation.

Deep-sequencing of 538 cancer-relevant genes (Table S1) was carried out in 57 HD-ALL patients lacking the common *KRAS* and *NRAS* codon 12 or 13 hotspot mutations and common B-cell ALL deletions. High quality sequencing data were obtained for each patient, with a mean coverage of 596X and an average of 94.3% reads with > 100X coverage. After removal of low quality variants, known polymorphisms, and likely germline mutations (based on mutant allele fraction, MAF), there were 108 variants predicted to be damaging (CADD Phred score ≥ 20) at 97 unique loci, including 90 SNVs and 7 INDELs (Figure 1). On average, patients harbored a median of only 1 mutation (range: 0-8) (Table S2), with no correlation between age-at-diagnosis and total number of mutations (R^2^ < 0.01). The most recurrently mutated genes included the RTK/Ras/MAPK signaling pathway genes *FLT3* (*n* = 12), *PTPN11* (*n* = 12), *KRAS* (*n* = 9), *NRAS* (*n* = 5), and the epigenetic regulator *CREBBP* (*n* = 5) (Figure 2). No novel hotspot mutations (*i.e.* recurrently mutated nucleotides) were identified. A descriptive summary of the predicted damaging mutation loci can be found in Table S2.

Based on DNA availability, we attempted to validate 62 mutations in recurrently mutated genes in the RTK/Ras/MAPK signaling pathway and involved in epigenetic regulation, including likely germline mutations. We successfully validated 60 out of 62 mutations (96.8%) in tumor DNA by PCR and Sanger sequencing. The 2 mutations that were not validated had MAF < 15% in the deep-sequencing, potentially explaining our inability to detect them *via* Sanger sequencing. Nineteen of the 62 mutations were also confirmed in matched germline DNA samples and, except for one mutation with MAF = 0.43, these had been predicted “likely germline” based on MAF.

### RTK/Ras/MAPK signaling pathway genes

In total, two-thirds (38/57) of HD-ALL patients carried a predicted damaging and somatic mutation in at least one RTK/Ras/MAPK signaling pathway gene (Figure S1). Twelve patients harbored a total of 15 mutations in *FLT3*, including 12 point mutations, one in-frame deletion of codon I836, and 2 internal tandem duplications (ITD) at the juxta-membrane region in exon 14. Six of the point mutations detected within *FLT3* were novel mutations in ALL (Figure S2), of which 2 (I867N and M664I) were in codons in which different amino acid-altering mutations were recently identified in HD-ALL [17], with the remaining 4 located in codons known to be mutated in AML. These novel mutations all lie within known mutational hotspot regions within or just upstream of the tyrosine kinase domain. In addition, we identified a *FLT3* mutation Y842C with MAF = 0.49 that was confirmed as a germline mutation *via* Sanger sequencing of remission DNA (Figure S2).

In *PTPN11*, one of the 9 mutation loci (F71L) was previously found in AML but not ALL according to COSMIC, though this mutation lies within one of the mutational hotspot regions in the N-terminal SH2 domain (Figure S3). The mutation P491L had MAF = 0.49 and was possibly germline, however constitutive DNA was not available for this patient to confirm this. In *KRAS*, we identified mutations at codon 146 (*n* = 4) and codon 117 (*n* = 1) (Figure 2, Figure S4). Subclonal mutations (MAF < 0.30) were also detected at codons 12 and 13 in 4 patients. In *NRAS*, mutations were found at known ALL hotspots at codons 61 (*n* = 4) and 146 (*n* = 1) (Figure S5). Additional likely-somatic damaging mutations were identified in the *Ras* homolog gene *MRAS* (*n* = 1), the leukemia oncogene *CBL* (*n* = 1), the *MYC* oncogene (*n* = 1), and in the receptor tyrosine kinase genes *ROS1* (*n* = 2), *ERBB2* (*n* = 1), *EPHB1* (*n* = 1), *FGFR1* (*n* = 1), *FGFR4* (*n* = 1), and *IGF1R* (*n* = 1). Mutations in *KRAS*, *NRAS*, *FLT3*, and *PTPN11* were largely mutually exclusive, with 38 mutations detected across 34 patients compared with a predicted distribution of 26±3.7 based on 10,000 permutations (*p* = 1.4×10^−5^; Z-score = 4.18). Of 38 patients with RTK/Ras/MAPK signaling pathway mutations, there were a total of 52 unique mutations. These were significantly mutually exclusive, with a distribution of 38 compared with a predicted distribution of 29.9±4.6 (*p* = 8.2×10^−5^; Z-score = 3.77) (Figure S1).

### Epigenetic regulatory genes

**Figure 3:**
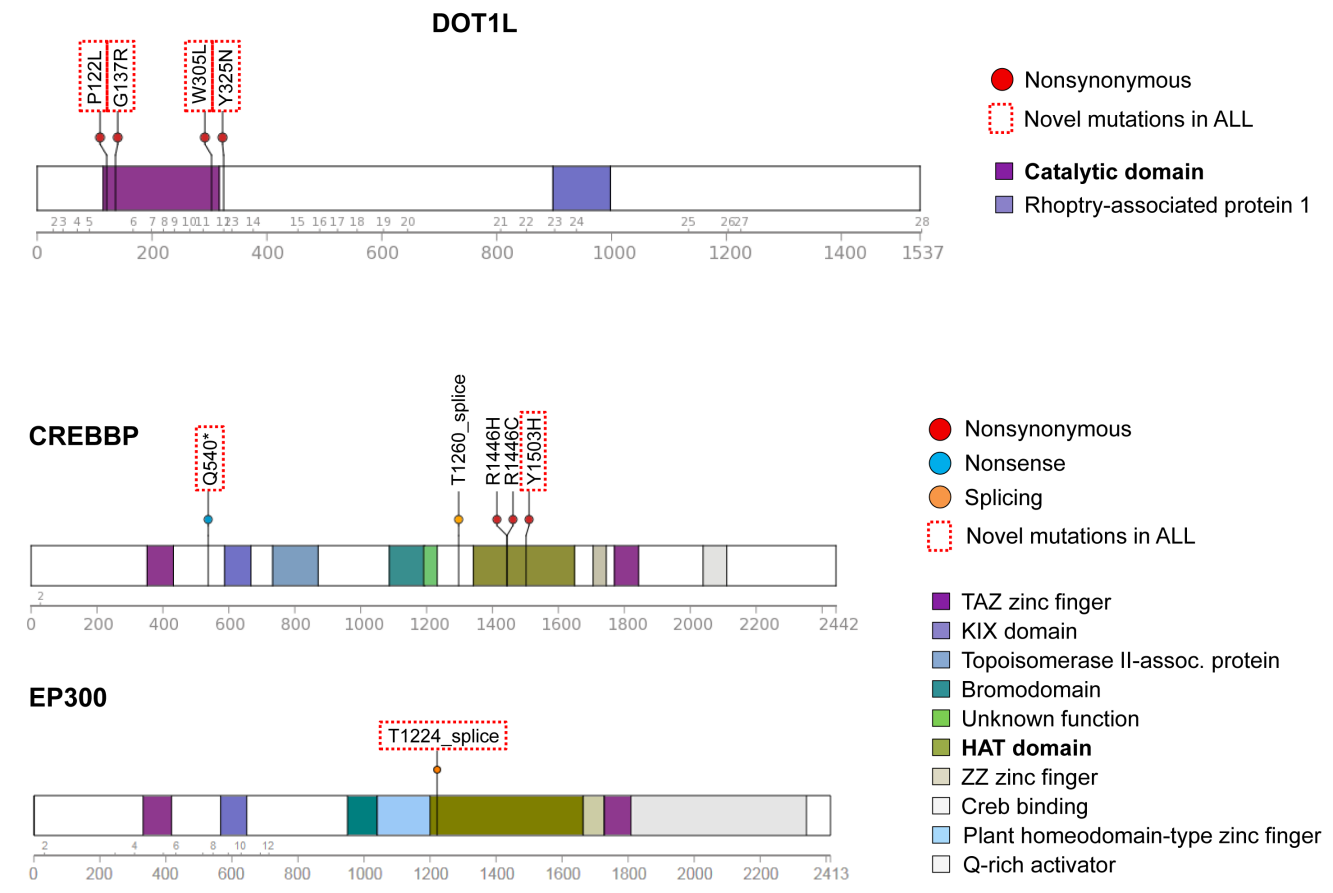
Novel mutations in epigenetic regulatory genes Schematic diagrams of DOT1L, CREBBP, and EP300 proteins showing positions of detected mutations in HD-ALL patients. Types of mutation (i.e. nonsynonymous, nonsense or splicing) are shown, along with color-coded functional domains as determined using the Protein Painter tool (http://explore.pediatriccancergenomeproject.org/proteinPainter). Bolded functional domains are ones affected by mutations. In DOT1L, nonsynonymous mutations cluster at the AdoMet catalytic domain. In CREBBP, nonsynonymous mutations cluster at the histone acetyltransferase (HAT) domain. Red dotted line boxes highlight novel mutations not previously reported in ALL.

Predicted damaging and somatic mutations in epigenetic regulators were detected in one-third (19/57) of HD-ALL patients (Figure S6). *CREBBP* mutations were the most frequent (*n* = 5), including previously unreported nonsense and missense mutations in the histone acetyltransferase (HAT) domain. Another patient had a splicing mutation within the HAT domain in the closely related gene *EP300* (Figure 3). *DOT1L* was also recurrently mutated (*n* = 4), including 3 mutations in the highly-conserved S-adenosylmethionine (AdoMet)-dependent methyltransferase domain with CADD Phred scores > 30, with an additional mutation adjacent to the AdoMet domain and also predicted to be highly deleterious (CADD Phred = 29.5) (Figure 3). Predicted damaging somatic mutations were also identified in *TRAPP* (*n* = 3) (Figure S7), and one each in *WHSC1*, *NCOR1*, *KMT2D*, *DNMT3A*, *EZH2*, *HIST1H1C*, *SETD2*, and *TAF1*. There was significant mutual exclusivity for epigenetic regulatory gene mutations, with 21 predicted damaging mutations detected across 19 patients compared with a predicted distribution of only 16.2±2.6 based on 10,000 permutations (*p* = 0.04; Z-score = 1.75) (Figure S6).

Epigenetic gene mutations were more frequent in patients with somatic *FLT3* mutations than in patients with *PTPN11* mutations, with 50% (6/12) of *FLT3* mutant patients harboring ≥ 1 epigenetic gene mutation, compared with only 9.1% (1/11) of patients with *PTPN11* mutations (*p* = 0.067, Fisher's exact test). *FLT3* mutations significantly co-occurred with *DOT1L* mutations, with 16 mutations across 13 patients (Figure 2) compared with a predicted distribution of 14.6±0.8 (*p* = 0.04; Z-score = −1.76). Further, *PTPN11* mutations were significantly mutually exclusive of epigenetic gene mutations (as a group), with 31 mutations across 29 patients compared with a predicted distribution of only 25.0±2.2 (*p* = 3.4×10^−3^; Z-score = 2.70). A similar trend was seen for *KRAS* (*p* = 0.12) and *NRAS* (*p* = 0.07) but not for *FLT3* (*p* = 0.49).

### Tumor microheterogeneity

**Figure 4:**
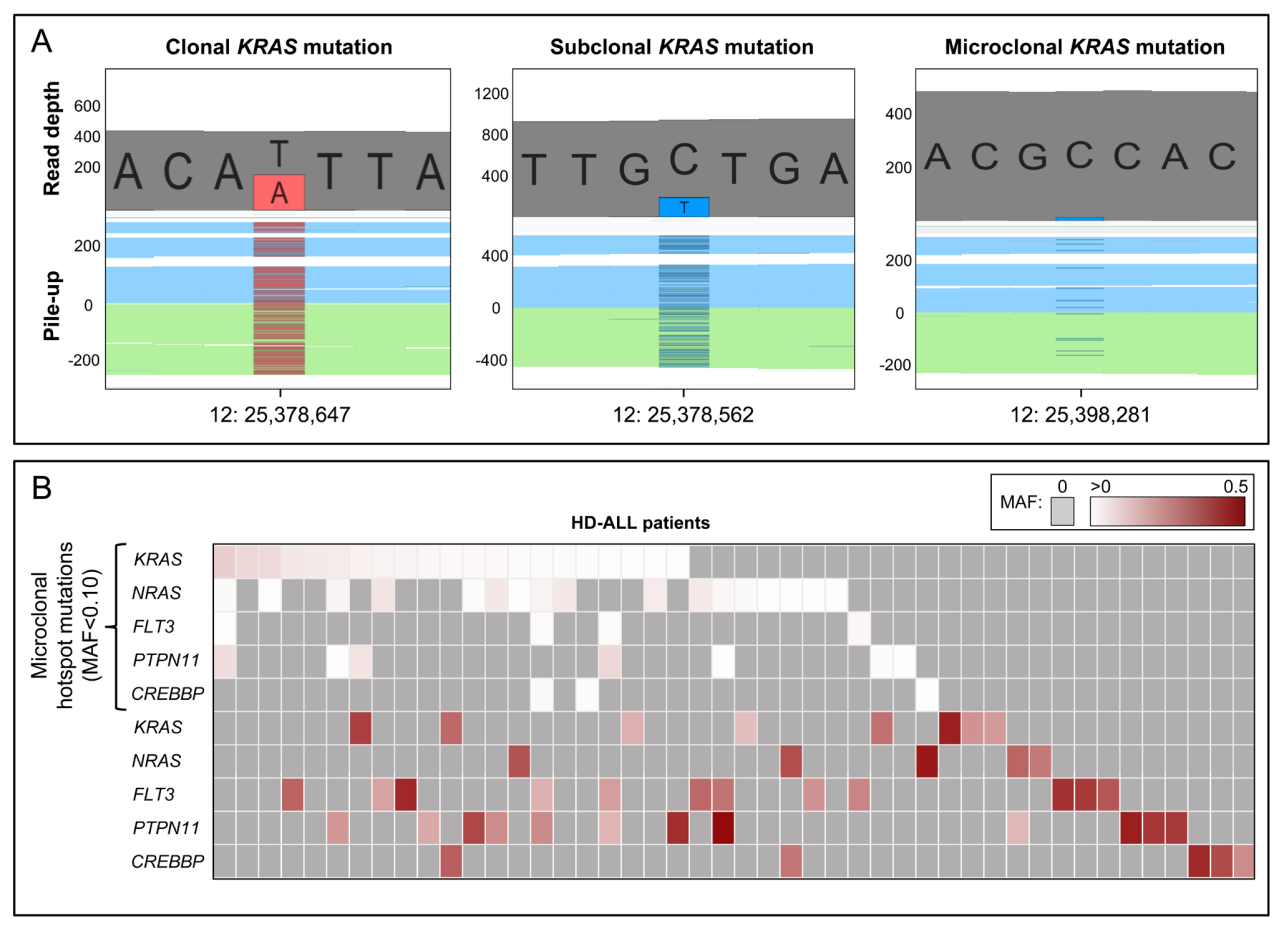
Clonal and microclonal tumor heterogeneity at HD-ALL hotspot loci **A.** GenomeBrowse screenshots showing clonal, subclonal, and microclonal *KRAS* mutations. In each screenshot, the top plot shows the total read depth and relative read depths of the reference (grey) and alternate alleles (red or blue). Bottom plots show the sequence read pile-up, split into forward (blue) and reverse (green) strands, with presence of mutant alleles shown in red or blue. The left screenshot shows a clonal *KRAS* T > A mutation at chr12:25,378,647 (K117N) with MAF = 0.44. The middle screenshot shows a subclonal *KRAS* C > T mutation at chr12:25,378,562 (A146T) with MAF = 0.20. The right screenshot shows a microclonal *KRAS* C > T mutation at chr12:25,398,281 (G13D) with MAF = 0.025. **B.** Heatmap showing the clonal and microclonal heterogeneity of HD-ALL hotspot mutations at *KRAS*, *NRAS*, *FLT3*, *PTPN11*, and *CREBBP*. The top 5 rows show the presence of microclonal (MAF < 0.10) hotspot mutations in codons 12, 13, 61, 117, and 146 of *KRAS* and *NRAS*; codon 835 of *FLT3*; codons 61 and 72 of *PTPN11*; and codon 1446 of *CREBBP*, across HD-ALL patients (columns). There were 21 patients with microclonal *KRAS* mutations (median MAF: 1.08%; range: 0.44-9.57%), 17 patients with microclonal *NRAS* mutations (median MAF: 1.28%; range: 0.39-5.47%), 4 patients with microclonal *FLT3* mutations (median MAF: 1.03%; range: 0.66-1.97%), 7 patients with microclonal *PTPN11* mutations (median MAF: 0.80%; range: 0.34-7.97%), and 3 patients with microclonal *CREBBP* mutations (median MAF: 0.68%; range: 0.48-1.13%). Clonal and subclonal mutations (MAF > 0.10) in the 5 genes are shown below, and include damaging mutations at additional codons. Mutations are color-coded according to their MAF as shown at the top right of the figure. Grey boxes represent patients with zero mutations. Where > 1 mutation was present in the same gene in a patient, the color of the cell represents the most clonal mutation. Patients without mutations in *KRAS*, *NRAS*, *FLT3*, *PTPN11*, or *CREBBP* were excluded from the figure.

We identified 65 minor subclonal, or “microclonal”, hotspot mutations at known mutation hotspot loci in *KRAS* (patient *n* = 21), *NRAS* (*n* = 17), *FLT3* (*n* = 4), *PTPN11* (*n* = 7), and *CREBBP* (*n* = 3) (Figure 4, Figure S8, Table S3). Median MAF of these mutations was 0.012 (range: 0.003-0.096). Of the 16 mutation loci assessed, only *FLT3* codon 663, *PTPN11* codon 69, and the negative control *NRAS* codon 117 did not harbor any microclonal mutations. Analysis of flanking sequences at the 65 microclonal hotspot mutations revealed only 5 additional mutations that qualified as microclonal in 3250bp (65 × 50bp) of sequence, thus 99.8% of flanking sequences did not harbor such mutations.

In total, there were 32 patients with microclonal mutations, of which 31 (54.4%) had at least one mutation in *KRAS*, *NRAS*, *FLT3*, and/or *PTPN11*. We assessed intra-sample tumor microheterogeneity and identified 16 patients with multiple microclonal mutations, including 10 patients carrying mutations in both *KRAS* and *NRAS* and 4 patients with mutations in at least 3 of the genes analyzed (Figure 4, Figure S8, Table S3). There was a highly significant co-occurrence of microclonal mutations in *KRAS* and *NRAS* codons 12 and 13, with 28 mutations found in only 19 patients compared with a predicted distribution of 23.8±2.1 (*p* = 4.8×10^−4^; Z-score = −3.3) (Figure S8). There was no significant difference between age-at-diagnosis in patients with microclonal hotspot mutations (mean = 4.67 yrs) and those without (mean = 4.89 yrs) (*p* = 0.77).

In addition, there were 8 patients with multiple microclonal mutations at the same hotspot locus, either in adjacent nucleotides within the same codon or in adjacent codons (Figure S9, Table S4). Five patients had adjacent *KRAS* hotspot mutations and 3 patients had adjacent *NRAS* mutations, including one patient with two codon 12 mutations (MAF = 2.9% and 5.1%) and one codon 13 mutation (MAF = 5.5%). In all 8 patients, these concurrent nucleotide changes occurred on different sequencing reads (*p* = 7.8×10^−3^, binomial significance test, 2-tailed), indicating that they were part of distinct tumor subclones (Table S4).

Including all identified clonal, subclonal, and microclonal mutations, there were 43 HD-ALL patients (75.4%) with a damaging mutation in *KRAS*, *NRAS*, *FLT3*, and/or *PTPN11*, and 8 patients (14.0%) with a damaging *CREBBP* mutation (Figure 4).

## DISCUSSION

Targeted deep-sequencing of over 500 cancer-relevant genes in 57 HD-ALL patients revealed extensive tumor heterogeneity in the RTK/Ras/MAPK signaling pathway, with the majority of patients harboring clonal, subclonal, and/or microclonal mutations at hotspot loci in *KRAS*, *NRAS*, *FLT3*, or *PTPN11*. Further, we found a substantial proportion of patients with epigenetic regulatory gene mutations, including in the putative novel driver gene *DOT1L*, and these typically co-occurred with mutations in *FLT3*.

We confirmed the high frequency and mutual exclusivity of mutations in *FLT3*, *PTPN11*, *KRAS*, and *NRAS* [13]. In *KRAS*, we verified that codon 146 and 117 mutations, recently identified by Paulsson *et al*. (2015) [16], are hotspot loci in HD-ALL. Indeed, 7 patients harbored microclonal mutations at one of these two loci (Figure S8). *FLT3* and *PTPN11* mutations were more frequent than previously reported, likely due to our patient selection criteria. We identified 6 novel mutations in *FLT3* and one novel mutation in *PTPN11*, in known activating hotspot regions but not previously detected in ALL. We also made the unexpected discovery of a likely causal germline *FLT3* mutation at Y842C. This same amino acid change was previously identified in tumor DNA from a non-hyperdiploid ALL patient [18], and was reported as an activating mutation in AML [19]. Recent analysis of germline mutations in childhood cancers identified a *FLT3* frameshift mutation in a child with E2A-fusion ALL [20]. However, to our knowledge this is the first report of a germline *FLT3* mutation in HD-ALL. Germline *PTPN11* mutations cause Noonan syndrome [21], therefore this patient may present with RASopathy-related phenotypes given that somatic mutations in *FLT3* and *PTPN11* activate the Ras/MAPK pathway and are largely mutually exclusive in ALL. However, clinical data were not available to confirm this.

Though we enriched for discovery of novel driver genes in HD-ALL, few novel recurrently mutated RTK/Ras/MAPK signaling genes were identified. Somatic alterations of *ROS1* have previously been detected in lung cancer and glioblastoma [22, 23], and we found *ROS1* mutations in a small fraction of HD-ALL patients. ROS1 protein mediates phosphorylation and increases activation of the *PTPN11*-encoded protein SHP2, and ROS1 activation can induce tumorigenesis [24]. Thus, the mutations we identified are potentially gain-of-function, suggesting that a subset of ALL patients could be treated with ROS1 inhibitors such as crizotinib [25] or foretinib [26].

The identification of subclonal *KRAS* codon 12 and 13 mutations in our initial analyses led us to investigate microclonal mutations (MAF < 10%) at ALL hotspot loci in *KRAS*, *NRAS*, *FLT3*, *PTPN11*, and *CREBBP*. Utilizing the high-coverage sequencing data we revealed an extraordinary level of intra- and inter-tumoral heterogeneity, with microclonal *Ras* pathway hotspot mutations detected in over 50% of patients. Adjacent microclonal mutations never appeared on the same sequencing reads and, therefore, appear to have arisen in distinct clones within tumor samples, supporting that the formation and selection of such hotspot mutations is ongoing. Further, the significant co-occurrence of *KRAS* and *NRAS* codon 12 and 13 microclonal mutations suggests the existence of a highly-heterogeneous subset of *Ras*-driven leukemias. Alternatively, these may not be driven by *Ras* pathway activation but by clonal mutations in other pathways that reduced the selective advantage of *Ras* mutant clones. These two possibilities are evidenced by a lack of clonal RTK/Ras/MAPK signaling gene mutations in the 9 patients with concurrent microclonal *Ras* codon 12/13 mutations, which included 2 patients harboring microclonal mutations at 4 different loci (Figure S8).

Microclonal *Ras* mutations have previously been reported in ALL and other tumor types [27, 28]. In a study of relapsed ALL, microclonal *KRAS* and *NRAS* mutations were detected in diagnostic DNA in over 50% of patients found to be wildtype by Sanger sequencing [27]. These patients had *Ras* mutations at relapse, suggesting that the microclonal mutations evaded initial treatment and drove the relapsed ALL [27]. Similarly, *CREBBP* HAT domain mutations are enriched in relapsed HD-ALL [17, 29-31]. Whether microclonal *Ras* or *CREBBP* mutations confer a similar risk of relapse to the more clonal mutations remains to be determined.

Mutations in epigenetic regulatory genes comprise a substantial portion of the mutational landscape of several hematologic malignancies, including T-ALL [32], AML [33], and B-cell lymphomas (reviewed in Lunning and Green [34]). We identified a high frequency and mutual exclusivity of epigenetic regulator mutations in HD-ALL, supporting a biological role for epigenetic dysregulation in this leukemia subtype [35] and that multiple possible genetic lesions may generate this disruption. Clustered damaging mutations discovered in the histone 3 lysine 79 (H3K79) methyltransferase gene *DOT1L* strongly support this as a driver of leukemogenesis. The only previously reported *DOT1L* mutation in childhood ALL was also in a HD patient, with a V114F mutation in close proximity to the AdoMet domain [36], suggesting *DOT1L* mutations may be specific to the HD subtype. In infant ALL, MLL-fusion proteins recruit DOT1L protein leading to H3K79 hypermethylation and increased expression of MLL target genes [37]. In contrast, DOT1L deficiency is associated with hyperploidy *in vitro*, likely due to aberrant mitotic spindle formation [38-40]. In mice, DOT1L knockout resulted in downregulation of GATA2 and differentiation towards the myeloid lineage during hematopoiesis [41]. We recently proposed that heritable risk alleles in *CEBPE*, a modulator of myelopoiesis, may increase ALL risk *via* lineage confusion [11]. Polymorphisms in *CEBPE* were more strongly associated with HD-ALL, supporting that de-differentiation of pre-B cells towards the myeloid lineage may be a hallmark of hyperdiploidy. *DOT1L* is also thought to play a role in double-strand break DNA repair and cell cycle control [42], thus more research is required to elucidate the role of *DOT1L* in HD-ALL.

Recent studies have shown that mutations in other epigenetic regulator genes, in particular the histone acetyltransferase *CREBBP*, are common in childhood ALL [16, 17]. We identified novel nonsense and HAT domain mutations in *CREBBP*, one of which (Y1503H) was previously reported in lymphoma [43]. Mutations in the H3K36 methyltransferase gene *WHSC1* were also recently reported in HD-ALL [16, 17]. In our patients, we did not detect the SET domain hotspot mutation at E1099 [16] but did find another SET domain mutation T1150A that was recently reported in B-ALL [30] and shown to have increased methyltransferase activity in mantle cell lymphoma [44]. We also identified novel mutations in *TRRAP*, a component of HAT complexes.

Only a fraction of known epigenetic regulatory genes were sequenced, thus it is likely that a higher proportion of HD-ALL tumors may suffer epigenetic perturbation. A recent study of over 1000 pediatric cancer genomes, including leukemias but not HD-ALL, revealed significant variation in frequency of epigenetic regulator mutations across tumor types [45]. Our data suggest that epigenetic dysregulation may be prominent in HD-ALL. One explanation is that hyperdiploidy leads to overexpression of tumor suppressor genes, which requires downregulation *via* epigenetic silencing. Indeed, some genes positioned on gained chromosomes in HD-ALL show decreased expression that is associated with hypermethylation [46, 47]. Alternatively, epigenetic dysregulation may be required for oncogene overexpression, supported by lower mean methylation levels in HD-ALL relative to other subtypes [46, 48], in particular on gained chromosomes [48, 49]. Drugs targeting epigenetic modifications, such as histone deacetylase (HDAC) inhibitors [50], are being explored as treatment options in ALL and might prove particularly effective against the HD-ALL subtype.

It was interesting to note that *DOT1L* mutations significantly co-occurred with mutations in *FLT3*. Moreover, epigenetic regulatory gene mutations were more frequent in patients harboring *FLT3* mutations than in patients with *PTPN11*, *KRAS*, or *NRAS* mutations, which tended to be mutually exclusive of epigenetic mutations. These could be a chance finding given the relatively small numbers, although *FLT3* mutations in AML frequently co-occur and functionally cooperate with mutations in epigenetic regulatory genes [33, 51-53]. Future studies should investigate whether *FLT3* activation cooperates with epigenetic dysregulation in ALL, which might suggest a potential use for combination treatment using FLT3 inhibitors with epigenetic therapies, as recently proposed for AML [54].

There are some caveats to consider when interpreting results from this study. First, the sequencing was limited to 538 genes included in the UCSF500 Cancer Gene panel and, though these are thought to be the most frequently mutated cancer genes, we may have missed potential driver mutations in genes not included on the panel. However, recent whole-genome and exome sequencing studies of HD-ALL did not identify any driver mutations in genes missing from our analyses [16, 17]. Clinical information, such as rate of relapse and outcome data, was unfortunately not available for the patients in this study. In addition, the exclusion of patients harboring common gene deletions precluded analysis of whether copy number alterations co-operate with mutations in *Ras* pathway or epigenetic regulatory genes in ALL. Therefore, next-generation sequencing of large numbers of unselected HD-ALL patients with available clinical data will be required to elucidate all possible leukemogenic pathways and the implications of particular clonal and microclonal mutations on patient outcomes.

### Conclusions

Deep-sequencing of HD-ALL genomes has revealed extensive tumor heterogeneity at both the clonal and microclonal level, highlighting the importance of RTK/Ras/MAPK signaling pathway activation and epigenetic dysregulation in HD leukemogenesis. The identification of putative novel driver genes *DOT1L* and *ROS1* demands further investigation into potential novel and targeted treatment strategies, such as ROS1 inhibition [26]. Targeting epigenetic modifications has been suggested as a new therapeutic option in ALL [30, 50]. Moreover, future work is required to investigate whether tumor microheterogeneity should impact therapeutic regimens in childhood ALL.

## MATERIALS AND METHODS

### Ethics statement

This study was reviewed and approved by institutional review committees at the University of California Berkeley, the California Department of Public Health (CDPH), and all collaborating institutions. Written informed consent was obtained from all parents of participants.

### Study subjects

Subjects included in this study were enrolled in the California Childhood Leukemia Study (CCLS) as previously described [55]. The initial set of patients comprised 457 newly-diagnosed B-ALL patients with cytogenetic subtype information, who were born in California and had diagnostic bone marrow samples available. These included 146 HD-ALL patients, of which 52.6% of patients were Hispanic and 33.6% were non-Hispanic white (the remainder included a mixture of African-American, Asian, and other ethnicities). Median age-at-diagnosis was 4.12 years (range 1.1-13.5). DNA was extracted from leukemia bone marrow collected at diagnosis using the QIAamp DNA Blood Mini Kit (QIAGEN, Germany).

### Determination of immuno-phenotype and cytogenetic profiles

Immuno-phenotype was determined for ALL patients using flow cytometry profiles, with those expressing CD10 or CD19 ( ≥ 20%) classified as B-lineage ALL, as described previously [56]. Ploidy was determined using FISH or G-banding, with HD patients classed as having > 50 chromosomes.

### Targeted sequencing of cancer genes

A summary of subject selection is included in the Supplementary Methods. In brief, 57 HD B-ALL patients with neither *Ras* codon 12/13 hotspot mutations nor common ALL deletions, as assessed by Sanger sequencing and multiplex ligation-dependent probe amplification respectively, were selected for deep-sequencing of cancer-relevant genes to enrich for discovery of novel driver mutations (Figure 1). NimbleGen SeqCap EZ libraries were used for capture of 538 genes included in the “UCSF500 Cancer Gene Panel” (Table S1). Library preparation was carried out according to manufacturer's protocol. DNA samples were barcoded and pooled for multiplexed sequencing on the Illumina HiSeq 2500 platform, with sequencing carried out to ~600X depth. Sequencing data analysis and variant filtering for predicted damaging SNVs and INDELs are described in detail in the Supplementary Methods and summarized in Figure 1. Briefly, to exclude likely germline polymorphisms we filtered out variants present in dbSNP or with a minor allele frequency > 0.01% (*i.e.* > 0.0001) in the Exome Aggregation Consortium (ExAC) Database. Predicted damaging mutations were identified using the Combined Annotation Dependent Depletion (CADD) tool version 1.3 (http://cadd.gs.washington.edu/score) [57]. A CADD Phred score threshold of ≥ 20 (*i.e.* top 1% deleterious variants in the genome) was used, as recommended for discovery of causal variants.

Given that targeted deep-sequencing data was not created from matched germline DNA samples, our variant filtering methods would not exclude rare germline variants. Thus, we used a mutant allele fraction (MAF) threshold of ≥ 0.45 to define “likely germline” variants. Due to the high frequency of chromosomal gains in HD-ALL, chromosome copy number must be considered when assessing clonality of mutations. For each patient, chromosomal copy number was inferred from sequencing data using the CNVkit software [58]. Whole chromosome gains were determined using mean log2 copy ratio and visual confirmation of copy number scatter plots. We then adjusted the MAF of each mutation based on copy number of the chromosome carrying that mutation, and based on the assumption that chromosomal gains occurred prior to development of somatic mutations. Hence, a mutation with MAF = 0.33 that was located on a chromosome with copy number of 3 would have an adjusted MAF of ~0.49 and, thus, was likely a germline variant that was present on a non-gained chromosome.

Sanger sequencing was carried out to validate a subset of mutations of interest (see Supplementary Methods and Table S2). Where available, remission DNA or neonatal bloodspot DNA was used to determine whether mutations were germline or somatic. Mutations were assessed in the Catalogue of Somatic Mutations in Cancer (COSMIC, http://cancer.sanger.ac.uk) [59] and in data from two recent sequencing studies of HD-ALL [16, 17], to determine whether they were previously identified in ALL or other cancer types. Clonal mutations were defined as those with MAF ≥ 0.30, and subclonal mutations with MAF ≥ 0.10 and < 0.30, following adjustment for chromosome copy number. Minor subclonal (*i.e.* microclonal) mutations were defined as having MAF < 0.10.

### Mutual exclusivity analysis

Mutual exclusivity analysis was carried out using Gitools (version 2.2.3) [60]. We included all genes in which ≥ 1 patient carried a predicted damaging somatic mutation. Mutation data were imported for all 57 patients, with a linear score corresponding to the mutant allele fraction as calculated from the reference:alternative allele ratio. Six patients carried 2 mutations in the same gene, and these mutations were grouped for each patient. Mutual exclusion analysis was carried out for genes in the RTK/Ras/MAPK signaling pathway and for those involved in epigenetic regulation, as well as for microclonal mutations. Observed distribution of mutations across patients (*i.e.* number of columns with a mutation) was compared to the expected distribution, and a Z-score obtained through 10,000 permutations, maintaining the number of “events” (*i.e.* mutations) per row and with weighted permutations for columns, as developed by Perez-Llamas, *et al*. [60]. P-values reflect the significance of mutual exclusivity derived from Z-scores.

### Tumor microheterogeneity analysis

To investigate tumor microheterogeneity, we assessed the frequency of microclonal mutations (*i.e.* MAF < 0.10). In this analysis, we included 15 known ALL hotspot loci and/or loci found to be recurrently mutated ( ≥ 1 patient) in our initial analysis of VCF data, including: *KRAS* codons 12, 13, 61, 117, and 146; *NRAS* codons 12, 13, 61, and 146; *FLT3* codons 663 and 835; *PTPN11* codons 61, 69, and 72; and *CREBBP* codon 1446. We also included *NRAS* codon 117 as a negative control, as this is not known to be mutated in ALL. Each patient BAM file was imported into the GenomeBrowse software (Golden Helix), and visual examination of the hotspot mutation loci was carried out. Inclusion criteria for microclonal mutations were nucleotide changes that were: 1) Nonsynonymous; 2) Present in ≥ 3 reads (*i.e.* > approximately 0.5% reads); 3) Present in both forward and reverse sequencing directions; and 4) Reads with mean base Phred quality score ≥ 30. To assess the validity of our methods, we counted the number of “non-hotspot” microclonal mutations in the 50bp immediately flanking each microclonal hotspot mutation (25bp up-/downstream) in patients harboring these mutations, relative to the total number of basepairs examined.

We observed several instances where ≥ 2 microclonal mutations in the same patient were present at adjacent nucleotides and/or in adjacent codons at the *KRAS* or *NRAS* codon 12/13 locus. To determine whether these mutations were likely in the same tumor clone or not, we exported sequencing read data and examined whether the adjacent nucleotide changes were present in the same reads or in different reads.

## SUPPLEMENTARY MATERIALS METHODS FIGURES AND TABLES










