# Supplementary material for: Clonal and microclonal mutational heterogeneity in high hyperdiploid acute lymphoblastic leukemia
Source: Oncotarget. 2016 Sep 24;7(45):72733–45. doi: 10.18632/oncotarget.12238 (PMC5341940; doi:10.18632/oncotarget.12238)
Supplement: Supplementary file 1 [file oncotarget-07-72733-s001.pdf]

Clonal and microclonal mutational heterogeneity in high hyperdiploid acute lymphoblastic leukemia

Supplementary Material

Subject selection for sequencing

KRAS and *NRAS* codon 12 and 13 hotspot mutation screening was carried out by Sanger sequencing as previously described [1]. In addition, multiplex ligation-dependent probe amplification (MLPA) was used to assess copy number at 8 genes commonly deleted in childhood ALL (*CDKN2A/B*, *IKZF1*, *PAX5*, *ETV6*, *RB1*, *BTG1*, *EBF1*, and the PAR1 region), using the MLPA probemix P335-B1 ALL-IKZF1 (MRC Holland) [2]. MLPA reactions and analyses were performed as previously described [1].

In the 146 HeH ALL cases, we found *KRAS* or *NRAS* hotspot mutations in 46 (31.5%) cases, and identified somatic loss of at least one of 8 genes tested in 51/146 (34.9%) cases. There were 64 (43.8%) HeH B-ALL cases with neither *Ras* hotspot mutations nor common deletions, of which sufficient DNA (>100ng) for targeted deep sequencing was remaining for 57 cases (Figure 1).

Sequence data analysis

Initial alignment of paired-end sequencing reads to the human reference genome (UCSC version hg19) was performed using the Burrows-Wheeler Aligner (BWA version 0.7.10-r789) [3], with reads sorted by position and converted to compressed BAM format using SAMtools [4]. Likely PCR and optical duplicate read pairs were marked using the Picard (version 1.97(1504)) command "MarkDuplicates" and removed using SAMBAMBA (version 0.4.7). Insertion and deletion (INDEL) realignment and recalibration were carried out using the Genome Analysis

Toolkit (GATK) [5]. Single nucleotide variants (SNVs) and small sequence INDELs were called in each sample using the GATK command “Unified Genotyper”, with variant calls stored in VCF files.

Variant filtering

VCF files were imported into the “SNP & Variation Suite” (SVS) software (Golden Helix, Inc., www.goldenhelix.com). Only variants with “PASS” in the “FILTER” tab of the VCF files were imported. SVS software includes multiple analysis tools for next-generation sequencing data. Figure 1 summarizes our pipeline for filtering of predicted damaging mutations. In brief, SNVs and INDELs were filtered based on low Read Depth (<10) and poor Genotype Quality scores (<15) to remove likely false positive calls, and then filtered against publicly available databases to remove likely germline polymorphisms. Any variants present in dbSNP human Build 141 (NCBI_2014_09_12_GrCh_37_g1k_Homo_sapiens) or with a minor allele frequency > 0.01% in the Exome Aggregation Consortium (ExAC) Dabatase (version 0.3) were removed (http://dx.doi.org/10.1101/030338). For variants present in ExAC with allele frequency <0.01%, we subsequently removed any of these variants with allele frequency >0.01% in the NHLBI (National Heart, Lung, and Blood Institute) Exome Sequencing Project using the Exome Variant Server (http://evs.gs.washington.edu/EVS/).

The Variant Classification tool was used in SVS to filter for coding variants, with only variants predicted to affect amino acid sequence (eg. nonsynonymous, stopgain, splicing) retained. To predict deleterious effects of variants, we used the Combined Annotation Dependent Depletion (CADD) tool version 1.3 (http://cadd.gs.washington.edu/score) [6], which integrates information from multiple functional annotation tools into a single score. As recommended for

discovery of causal variants, a CADD Phred score threshold of ≥20 (*i.e.* top 1% deleterious variants in the genome) was used. GenomeBrowse software (Golden Helix) was used for manual inspection of coding SNVs and INDELs in the tumor BAM files for additional screening of false positive calls, eg. variants showing strand bias. The GeneCards tool "GeneALaCart" (https://genealacart.genecards.org/) was used to retrieve information and assess function of genes included in the UCSF500 Cancer Gene Panel, to determine which cancer-related pathways were recurrently mutated.

Mutation validation

Sanger sequencing was used to validate a subset of mutations of interest (Table S2), including in recurrently mutated genes in the RTK/Ras/MAPK signaling pathway and involved in epigenetic regulation, including all mutations in the SWI/SNF chromatin remodeling complex. Where available, we assessed whether mutations were acquired in the tumor or were inherited, through sequencing of matched germline DNA samples. Germline DNA was available for 44 out of 57 patients, including from either buccal swabs (n=31) or saliva (n=3) acquired in remission, or from neonatal bloodspots (n=10). PCR primers were designed using Primer3 software (http://bioinfo.ut.ee/primer3/), and reactions carried out using the Advantage 2 PCR kit (Clontech). PCR products were cleaned up using ExoSAP-IT reagent (Affymetrix), and sequenced bi-directionally using an ABI 3730xl DNA sequencer. Sequence chromatogram files were analyzed using Chromas software (Technelysium).

References

1. Walsh KM, de Smith AJ, Welch TC, et al. Genomic ancestry and somatic alterations correlate with age at diagnosis in Hispanic children with B-cell acute lymphoblastic leukemia. Am J Hematol. 2014;89(7):721-725.

2. Schwab CJ, Chilton L, Morrison H, et al. Genes commonly deleted in childhood B-cell precursor acute lymphoblastic leukemia: association with cytogenetics and clinical features. Haematologica. 2013;98(7):1081-1088.

3. Li H and Durbin R. Fast and accurate short read alignment with Burrows-Wheeler transform. Bioinformatics. 2009;25(14):1754-1760.

4. Li H, Handsaker B, Wysoker A, et al. The Sequence Alignment/Map format and SAMtools. Bioinformatics. 2009;25(16):2078-2079.

5. McKenna A, Hanna M, Banks E, et al. The Genome Analysis Toolkit: a MapReduce framework for analyzing next-generation DNA sequencing data. Genome Res. 2010;20(9):1297-1303.

6. Kircher M, Witten DM, Jain P, O'Roak BJ, Cooper GM, Shendure J. A general framework for estimating the relative pathogenicity of human genetic variants. Nat Genet. 2014;46(3):310-315.

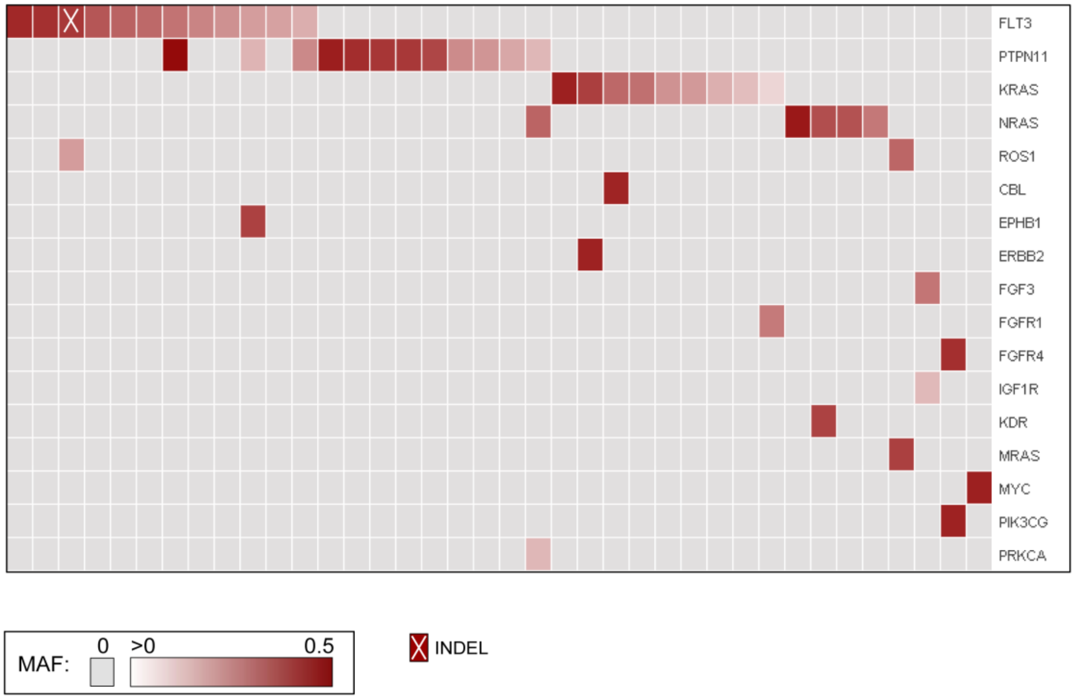

Figure S1 – Mutational spectrum of RTK/Ras/MAPK signaling genes in HD-ALL. Tiling plot includes predicted damaging somatic and likely somatic mutations in RTK/Ras/MAPK genes (rows) across HD-ALL patients (columns). Confirmed and likely germline mutations were excluded. Only patients with mutations in this pathway are included in this figure. Mutations are color-coded according to their mutant allele fraction (MAF), with MAF adjusted for chromosome copy number. Mutations were significantly mutually exclusive, with 52 mutations distributed across 38 patients compared with a predicted distribution of 29.9 ±4.6 based on permutations ($P=8.2x10^{-5}$; Z-score = 3.77).

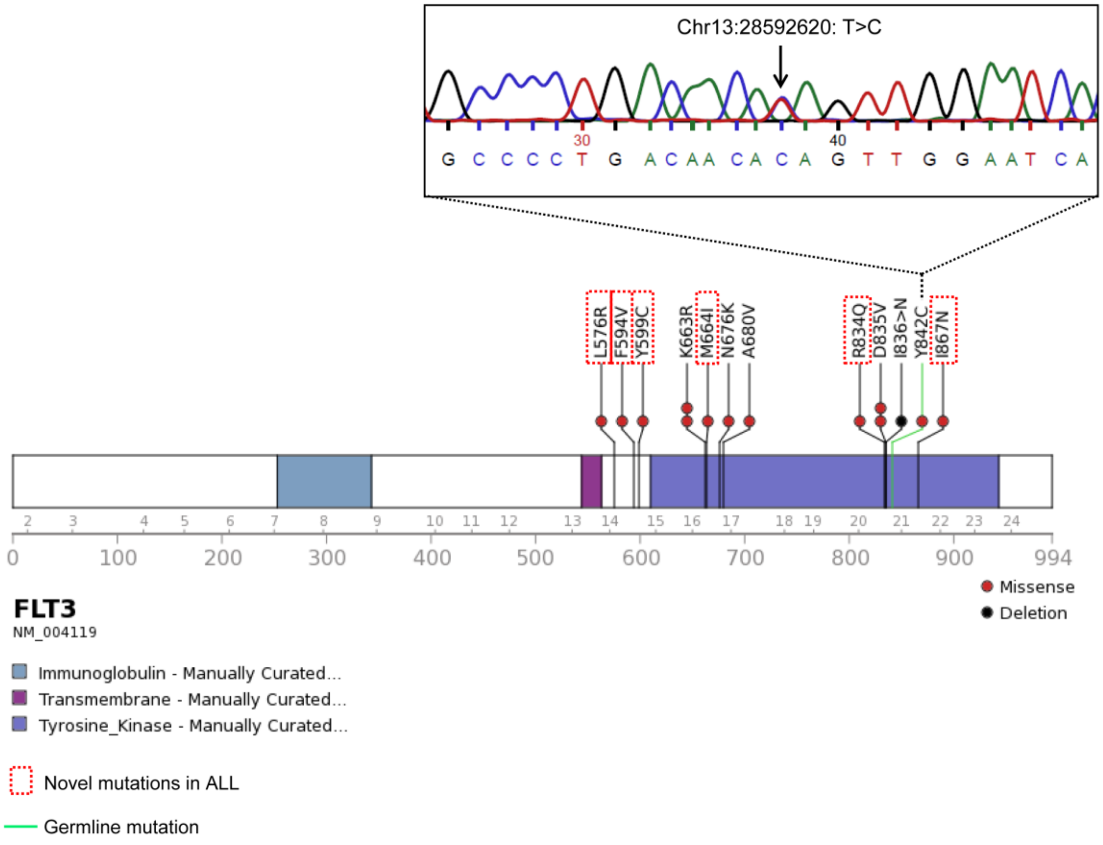

Figure S2 – Mutations in the RTK/Ras/MAPK signaling gene *FLT3*, including 6 novel ALL mutations at known hotspot loci. Color-coded functional domains illustrated using the Protein Painter tool (http://explore.pediatriccancergenomeproject.org/proteinPainter). The Y842C mutation was found to be present in the germline DNA of one patient, as highlighted by the sequence chromatogram at this locus.

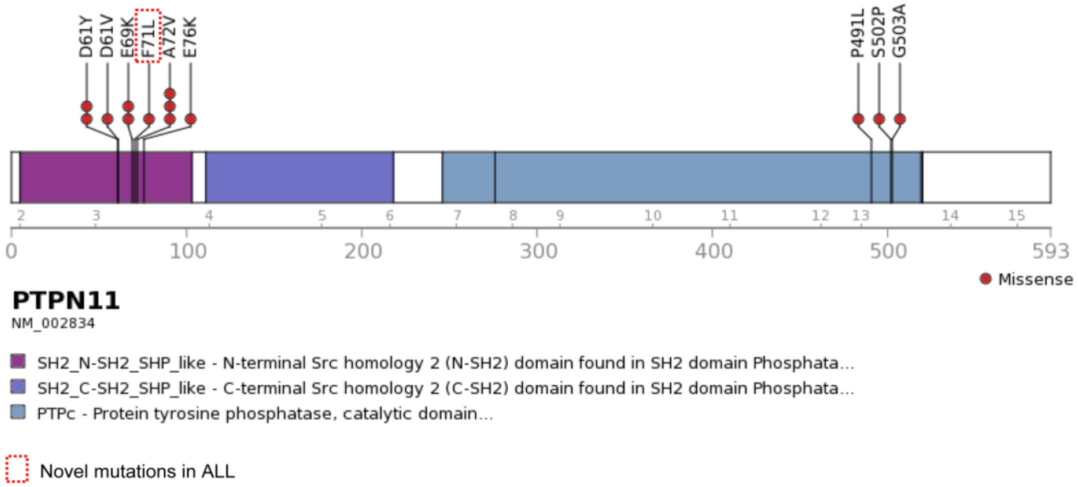

Figure S3 – Mutations in the RTK/Ras/MAPK signaling gene *PTPN11*, including 1 novel ALL mutation at a known ALL hotspot locus. Color-coded functional domains illustrated using the Protein Painter tool (http://explore.pediatriccancergenomeproject.org/proteinPainter).

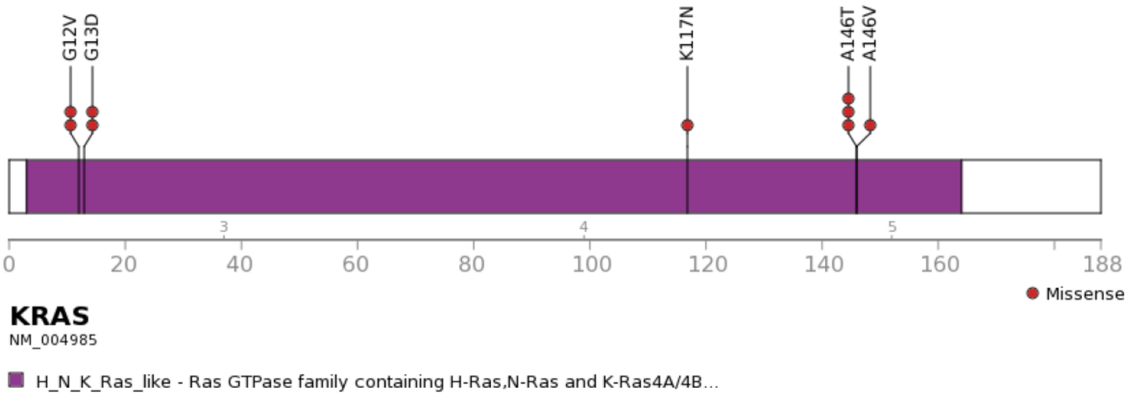

Figure S4 – Mutations in the RTK/Ras/MAPK signaling gene *KRAS*, at known hotspot loci. Color-coded functional domains illustrated using the Protein Painter tool (http://explore.pediatriccancergenomeproject.org/proteinPainter).

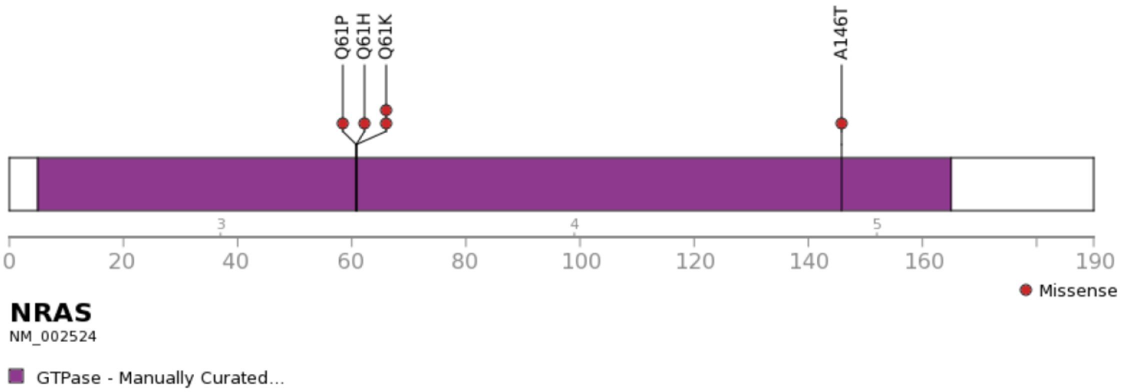

Figure S5 – Mutations in the RTK/Ras/MAPK signaling gene *NRAS*, at known hotspot loci. Color-coded functional domains illustrated using the Protein Painter tool (http://explore.pediatriccancergenomeproject.org/proteinPainter).

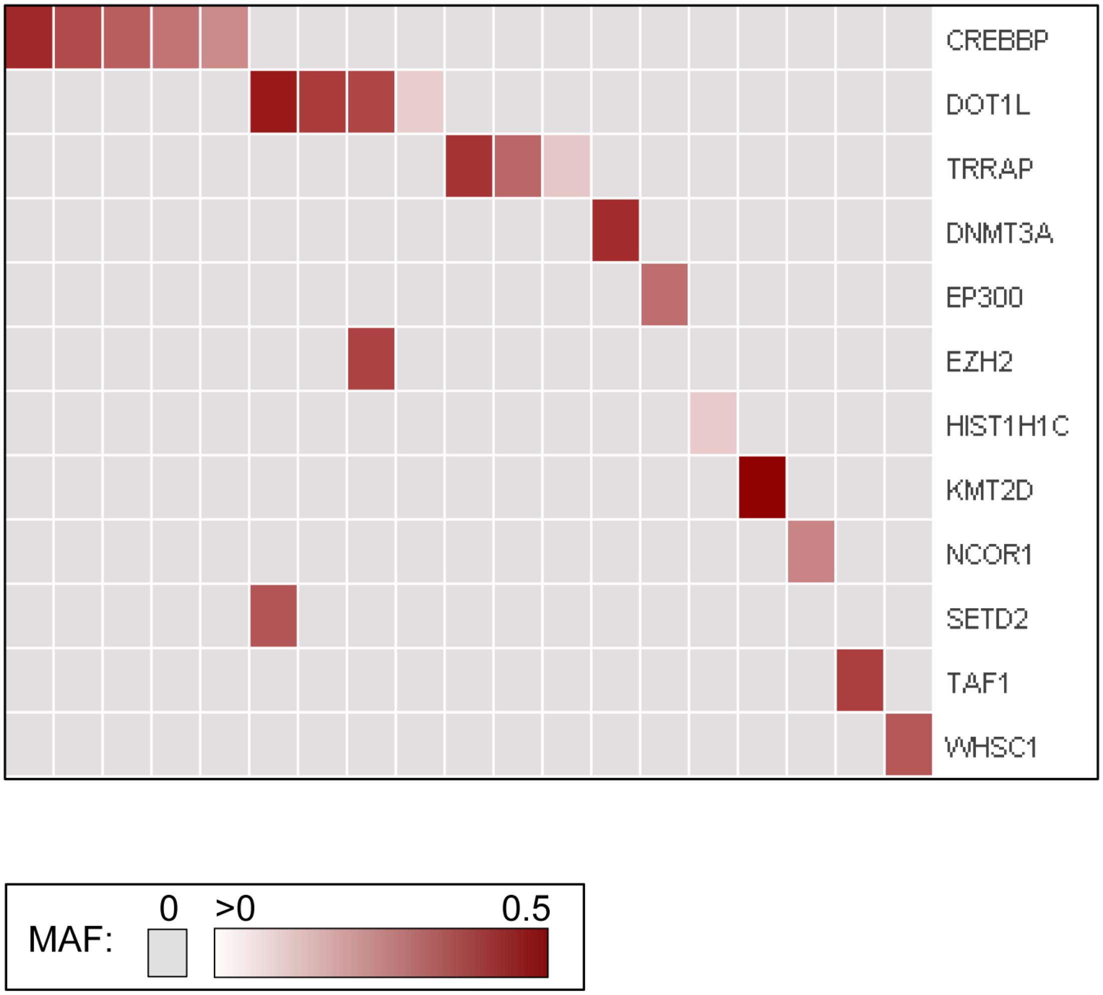

Figure S6 – Mutational spectrum of epigenetic regulatory genes in HD-ALL. Tiling plot includes predicted damaging mutations in all sequenced epigenetic regulatory genes (rows) across the HD-ALL patients (columns). Confirmed and likely germline mutations were excluded. Only patients with mutations in genes involved in epigenetic regulation are included in this figure. Mutations are color-coded according to their mutant allele fraction (MAF), with MAF adjusted for chromosome copy number. Mutations were significantly mutually exclusive, with 21 mutations distributed across 19 patients compared with a predicted distribution of 16.2 ±2.6 based on 10,000 permutations (P=0.04; Z-score = 1.75).

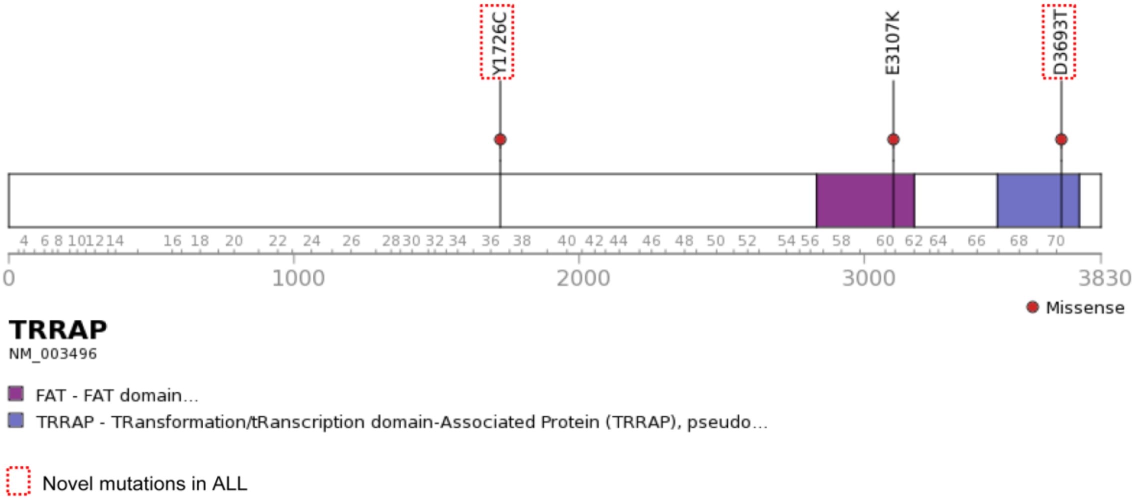

Figure S7 – Mutations in epigenetic regulatory gene *TRAPP* (Transformation/Transcription Domain-Associated Protein), including 2 novel ALL mutations at Y1726C and D3693T. Color-coded functional domains illustrated using the Protein Painter tool (http://explore.pediatriccancergenomeproject.org/proteinPainter). The known E3107K and novel D3693T mutations are located within the ATM-related FAT domain and the PI3-kinase domain respectively, both with CADD scores >30.

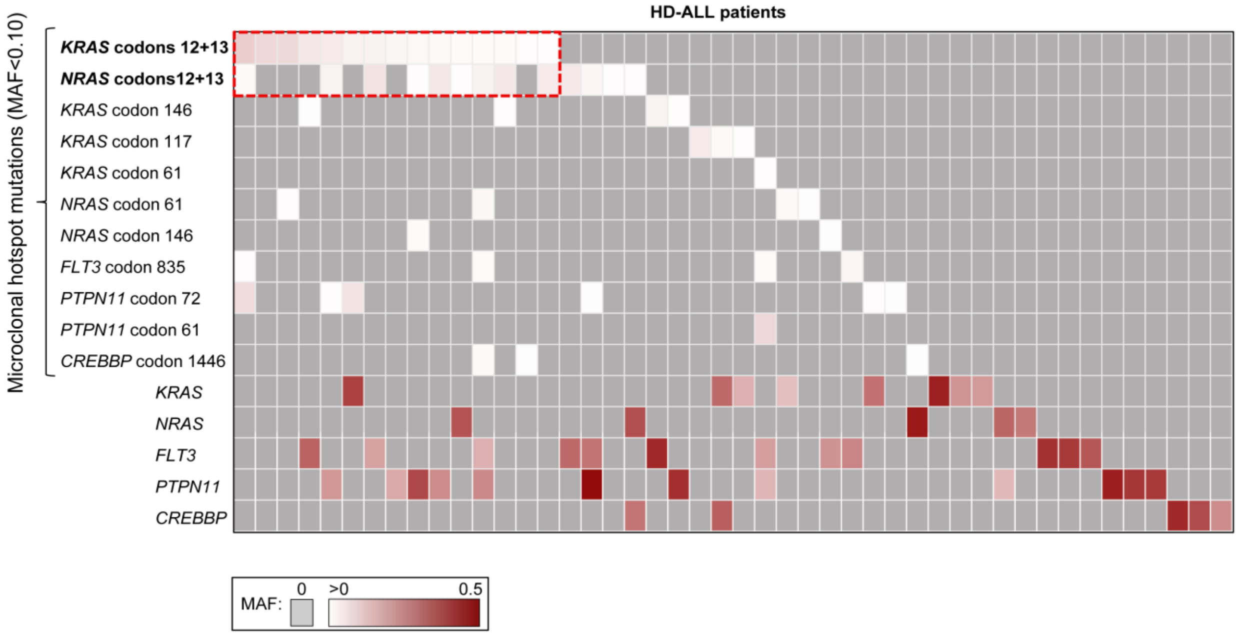

Figure S8 – Heatmap showing the clonal and microclonal heterogeneity of HD-ALL hotspot mutations at *KRAS*, *NRAS*, *FLT3*, *PTPN11*, and *CREBBP*. The first 11 rows show the presence of microclonal (MAF<0.10) mutations at hotspot loci in these 5 genes, across HD-ALL patients (columns). Red dotted box highlights the significant co-occurrence of microclonal mutations in *KRAS* and *NRAS* codons 12 and 13, with 28 mutations found in only 19 patients compared with a predicted distribution of 23.8±2.1 ($P=4.8x10^{-4}$; Z-score=-3.3). *NRAS* codon 117, *FLT3* codon 663, and *PTPN11* codon 69 were included in the analysis but not found to harbor microclonal mutations in any patients, thus are not included in the heatmap.

Clonal and subclonal mutations (MAF>0.10) in *KRAS*, *NRAS*, *FLT3*, *PTPN11*, and *CREBBP* are shown below. Mutations are color-coded according to their MAF as shown at the bottom of the figure. Grey boxes represent patients with zero mutations.

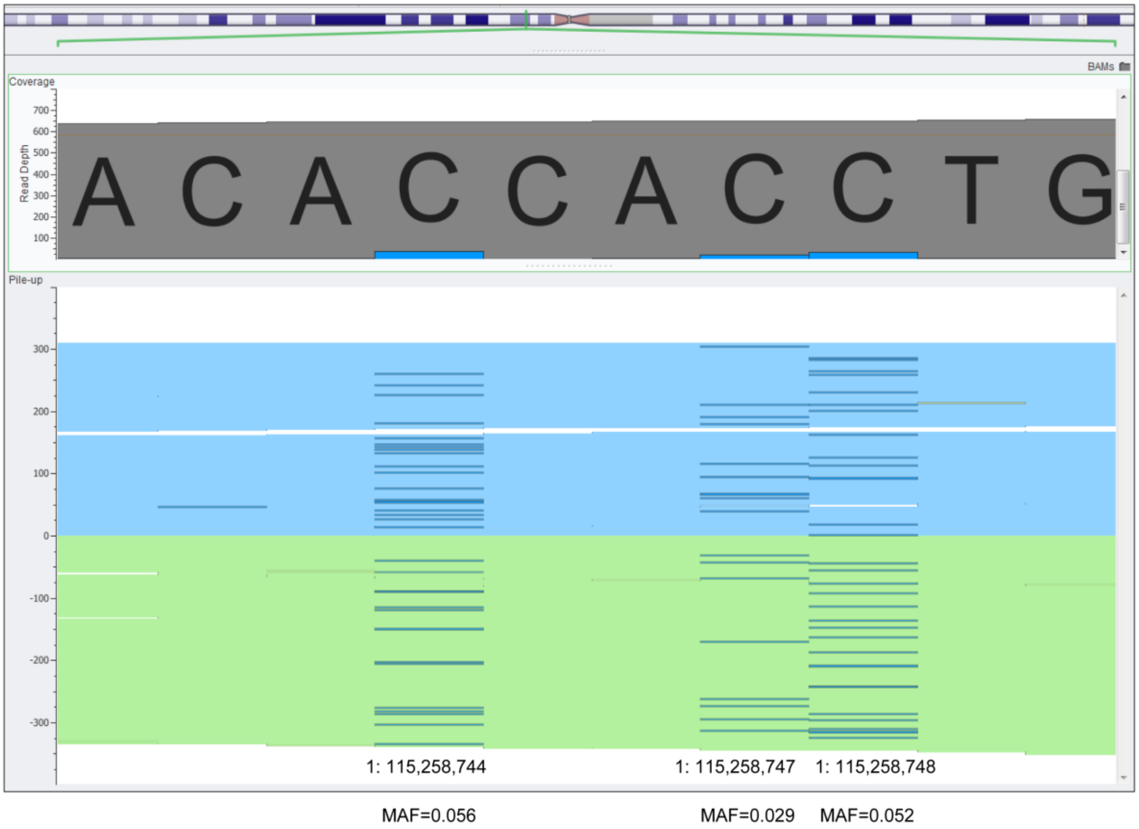

Figure S9 – GenomeBrowse plot showing microclonal (MAF<0.10) *NRAS* codon 12 and 13 mutations in the same tumor sample, at chr1:115258744, chr1:115258747, and chr1:115258748. The top plot shows the total read depth and relative read depths of the reference alleles C (grey) and the alternate T alleles (blue), which have MAFs = 0.056, 0.029, and 0.052 from left to right. The bottom plot shows the sequence read pile-up, split into forward (blue) and reverse (green) strands, with presence of mutant alleles highlighted in blue. Further analysis revealed the concurrent nucleotide changes occurred on different sequencing reads (Table S3).

Table S1 – List of genes included in the UCSF500 Cancer Gene Panel.

Table S2 – Summary of predicted damaging mutations identified in HD-ALL patients. Each row contains information corresponding to a mutation detected in one of 57 patients. Chromosome position is according to the hg19 (GRCh37) genome build. The amino acid coding changes are according to the Human Genome Variation Society (HGVS), for transcript 1 of each protein. Allele frequency (AF) of each mutation is shown according to the Exome Aggregation Consortium (ExAC) and the NHLBI (National Heart, Lung, and Blood Institute) Exome Sequencing Project.

Table S3 – Summary of microclonal mutations at hotspot loci in *KRAS, NRAS, FLT3, PTPN11*, and CREBBP. Each row contains information corresponding to a mutation detected in one of 57 patients. Chromosome position is according to the hg19 (GRCh37) genome build.

Table S4 – Mutual exclusivity of adjacent *Ras* hotspot microclonal (MAF<0.10) mutations.
